# GIP1 and GIP2 Contribute to the Maintenance of Genome Stability at the Nuclear Periphery

**DOI:** 10.3389/fpls.2021.804928

**Published:** 2022-01-27

**Authors:** Gaurav Singh, Morgane Batzenschlager, Denisa Tomkova, Etienne Herzog, Elise Hoffmann, Guy Houlné, Anne-Catherine Schmit, Alexandre Berr, Marie-Edith Chabouté

**Affiliations:** ^1^Institut de Biologie Moléculaire des Plantes, CNRS, Université de Strasbourg, Strasbourg, France; ^2^Cell Biology, Faculty of Biology, University of Freiburg, Freiburg, Germany

**Keywords:** *A. thaliana*, genome stability, root meristem, GIP, RAD51 foci, γ-H2AX foci

## Abstract

The maintenance of genetic information is important in eukaryotes notably through mechanisms occurring at the nuclear periphery where inner nuclear membrane proteins and nuclear pore-associated components are key factors regulating the DNA damage response (DDR). However, this aspect of DDR regulation is still poorly documented in plants. We addressed here how genomic stability is impaired in the *gamma-tubulin complex component 3-interacting protein* (*gip1gip2*) double mutants showing defective nuclear shaping. Using neutral comet assays for DNA double-strand breaks (DSBs) detection, we showed that GIP1 and GIP2 act redundantly to maintain genome stability. At the cellular level, γ-H2AX foci in *gip1gip2* were more abundant and heterogeneous in their size compared to wild-type (WT) in root meristematic nuclei, indicative of constitutive DNA damage. This was linked to a constitutive activation of the DDR in the *gip1gip2* mutant, with more emphasis on the homologous recombination (HR) repair pathway. In addition, we noticed the presence of numerous RAD51 foci which did not colocalize with γ-H2AX foci. The expression of GIP1-GFP in the double mutant rescued the cellular response to DNA damage, leading to the systematic colocalization of RAD51 and γ-H2AX foci. Interestingly, a significant proportion of RAD51 foci colocalized with GIP1-GFP at the nuclear periphery. Altogether, our data suggest that GIPs may partly contribute to the spatio-temporal recruitment of RAD51 at the nuclear periphery.

## Introduction

Safeguarding the genetic information is essential in cells under endogenous and exogenous stresses leading to DNA damage. The integrity of genetic information has also to be maintained in cycling cells during DNA replication and during mitosis. In eukaryotes, DNA lesions lead to the activation of specific networks of proteins which are recruited at DNA damage sites for signaling and repair.

Plants are constantly facing environmental stresses leading to various forms of DNA lesions, with DNA double-strand breaks (DSBs) as the most serious form of DNA damage (West et al., 2004). Besides their induction by exogenous genotoxic stresses, DSBs can also arise either from DNA replication defects such as stalling replication fork or as a result of an increased level of endogenous Reactive Oxygen Species (ROS). Un-repaired or mis-repaired DSBs in dividing cells can lead to the formation of aberrant chromosomes and thus to developmental defects (Roy, 2014). DSBs repair is mainly mediated by either Homologous Recombination (HR) or Non-homologous End Joining (NHEJ) (West et al., 2004). In this context, chromatin organization around damage sites plays also a crucial role in the DNA damage response (DDR) by providing a scaffold and/or easy access to the DNA repair machinery (Takatsuka et al., 2021). The γ-H2AX protein (a phosphorylated form of the histone variant H2AX) accumulates at DSBs (Friesner et al., 2005) and is instrumental for the recruitment of DNA repair signaling and repair factors (Lang et al., 2012; Amiard et al., 2013; Biedermann et al., 2017; Horvath et al., 2017). Besides the recruitment at DSBs of BREAST CANCER SUSCEPTIBILITY1 (BRCA1), RADIATION SENSITIVE51 (RAD51), and RAD54 which are involved in HR (Biedermann et al., 2017; Hirakawa et al., 2017; Horvath et al., 2017), the RETINOBLASTOMA-RELATED 1 (RBR1) and the transcription factors E2Fa are also found at DSBs to promote HR (Lang et al., 2012; Biedermann et al., 2017; Horvath et al., 2017; Raynaud and Nisa, 2020). More recently, the important cell-cycle regulatory protein F-BOX-LIKE17 (FBL17) was shown to be a regulator of the DDR and to colocalize as well with RBR1 and γ-H2AX at DNA lesions, suggesting connections between cell cycle and DDR in plants (Gentric et al., 2020, 2021).

In mammals the nucleo-cytoplasmic interface and its associated protein complexes, such as the nuclear pore complex (NPC), the LINC complexes (Linker of Nucleoskeleton and Cytoskeleton) or the nuclear lamina play critical roles in regulating DDR (Bukata et al., 2013; Ryu et al., 2015). However, such regulatory processes, in the context of the nuclear envelope (NE) environment, still remain poorly investigated in plants. The SAD1/UNC-84 (SUN1 and SUN2) domain proteins, belonging to the LINC complexes, at the inner nuclear membrane of the NE regulate meiotic recombination (Varas et al., 2015). RAD54, required for HR in somatic cells, forms foci in response to irradiation in *Arabidopsis* root cell nuclei that accumulate at the nuclear periphery (Hirakawa and Matsunaga, 2019). As components of the plant nucleoskeleton, CROWDED NUCLEI (CRWN) proteins protect genomic DNA against excessive oxidative damages caused by the DNA damaging agent methyl methanesulfonate (Wang et al., 2019). Finally, the HIGH EXPRESSION OF OSMOTICALLY RESPONSIVE GENE1 (HOS1), as part of the NPC (Cheng et al., 2020), was recently reported to activate DNA repair components in response to heat-induced DNA damages (Han et al., 2020).

Previously, we showed that the gamma-tubulin complex component 3-interacting proteins (GIPs), located on both side of the NE, are key players in regulating the plant nuclear architecture and organization, notably through the maintenance of centromeric cohesion at the nuclear periphery of Arabidopsis root meristem nuclei (Janski et al., 2012; Batzenschlager et al., 2013; Batzenschlager et al., 2015; Chabouté and Berr, 2016). In this work, using cellular and molecular experimental approaches, we detected numerous endogenous γ-H2AX foci, indicative of constitutive DSBs, as well as the preferential activation of the HR signaling pathway in *gip1gip2.* This might rely on defective HR repair linked to impaired colocalization of γ-H2AX with the DNA repair protein RAD51 involved in HR. However, upon genotoxic stress this colocalization is restored in the *gip1gip2* mutant complemented by the expression of a GIP1-GFP fusion protein. Finally, we also detected a partial but significant colocalization between GIP1-GFP and RAD51 foci at the nuclear periphery. Together, our findings shed light on the contribution of GIPs at the nuclear periphery for genome maintenance and proper localization of RAD51 foci.

## Materials and Methods

### Plants, Growth, and Treatment Conditions

The mutants *gip1, gip2*, *gip1gip2* and their corresponding genetic background Col-0, WS, and Col-0 x WS have been described previously (Janski et al., 2012; Batzenschlager et al., 2017). Arabidopsis seedlings were grown *in vitro* on ½ Murashige and Skoog medium (SERVA Electrophoresis) in presence of 1% sucrose supplemented with 1.2% agar at 20°C under long day conditions (16-h light 70 μmol m^–2^ s^–1^ of fluorescent lighting/8-h dark). The *gip1gip2* mutant was complemented by the expression of a pGIP1::GIP1-GFP construct as previously described (Janski et al., 2012; Batzenschlager et al., 2015). For sensitivity tests to genotoxic drugs, seeds were initially sowed on ½ MS agar and after 5 days on growth, seedlings were transferred on media supplemented with the genotoxins. Drug concentrations were 10 μM for Bleomycin (BLM) (Laboratoire Thissen, Belgium) and 50 μM for cisplatin (CP) (Sigma, St. Louis, United States). Seedlings were treated during 16 h by 50 μM CP in ½ MS for cytological analyses.

### Neutral Comet Assay

Nuclei were isolated from 9-day-old seedlings using a Chopping solution and comet assays were performed as described (Roa et al., 2009; Lang et al., 2012). The quantification of the comet figures was related to an arbitrary scoring of the comet figures as described previously (Collins, 2004). In each assay, 100 comets were scored and the results represent the mean values from three independent experiments.

### Immunostaining

Nine-day-old Arabidopsis seedlings were fixed in 4% PFA and processed as described previously on nuclei from squashed root tip (Batzenschlager et al., 2015). The primary anti-γ-H2AX antibody (diluted at 1/500) produced by Davids Biotechnology (Regensburg, Germany; Friesner et al., 2005), the rat anti-RAD51 antibody (diluted at 1/500) (Kerzendorfer et al., 2006) and when needed the monoclonal antibody directed against GFP (diluted at 1/500) (Invitrogen, Thermo Fisher Scientific) were incubated overnight at 4°C. Signals were revealed accordingly with the following secondary antibodies: Alexa fluor-488 goat anti-rabbit (diluted at 1/200), the Alexa fluor-488 goat anti-mouse (diluted at 1/200), the Alexa fluor-568 goat anti-rat (diluted at 1/300) and the Cy5 goat anti-rabbit (diluted at 1/300) (Life Technologies, Thermo Fisher Scientific). Root tips were mounted in antifade Vectashield (Vector Laboratories), with DAPI (2 μg/ml).

### Confocal Analyses

Confocal images were recorded with a Zeiss LSM 780 microscope equipped with an oil 63 × /1.4 NA lens. The excitation and emission wavelengths for Alexa 488 were 488 and 510 nm, respectively. To reveal Alexa 568, the excitation and emission wavelengths were 555 and 617 nm, respectively. For DAPI observations, the excitation and emission wavelengths were 405 and 500 nm, respectively. Cy5 was combined with DAPI, with excitation and emission wavelengths of 571 and 735 nm, respectively. Observations were performed in multi-tracking mode using 405-, 488-, or 561-nm laser excitation. Images were processed using the ImageJ software.

### RNA Extraction and Real-Time RT-qPCR

Total RNA was extracted from 9-day-old *Arabidopsis thaliana* seedlings using the Nucleospin RNA Plant kit (Macherey-Nagel, Hoerdt, France) according to manufacturer’s instructions after grinding with glass beads (1, 7/2 mm) in a Precellys^®^24 grinder (Bertin Technologies, Saint-Quentin-en-Yvelines, France) at 5,500 rpm, 2 × 30 s. For RT-qPCR, 2.5 μg of RNA were used to synthesize cDNA using specific primers, random hexamer primers (IDT) and the protocol “SuperScript^®^ IV (SSIV) First-strand and cDNA Synthesis Reaction” (Invitrogen). RT-qPCR was performed on a LightCycler 480 II (Roche) with SYBR Green Master Mix (Bio-Rad), according to the manufacturer’s instructions. All primers used are described in Supplementary Table 1. Quantification was done using the ΔΔCt method and normalized to ACTIN2 (Livak and Schmittgen, 2001).

## Results

### High Genomic Instability Is Linked to Endogenous Double-Strand Breaks in *gip1gip2*

**FIGURE 1:**
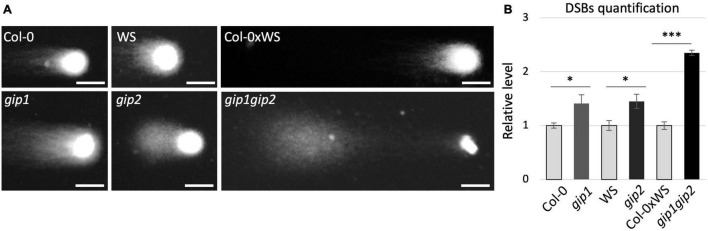
Genomic instability is high in the *gip1gip2* double mutant. **(A)** Nine-day-old Arabidopsis plantlets from single and double mutants (*gip1*, *gip2*, *gip1gip2*) and their respective WT backgrounds (Col-0, WS, and Col-0xWS) were used for neutral comet assay. Representative images of comet assay are presented. Scale bar = 5 μm. **(B)** The quantification of DSBs was performed on at least 100 nuclei by giving an arbitrary score to each comet figure as previously described (Lang et al., 2012). The Mann Whitney test was used and *p*-values are indicated as < 0.05 (*) and < 0.001 (^***^), respectively. The results were obtained from three independent experiments. Error bars indicate SEM.

Our previous data have shown that chromosome instability occurs during mitosis in the *gip1gip2* double mutant, resulting in the appearance of micronuclei in interphase cells and ploidy defects (Janski et al., 2012; Batzenschlager et al., 2015). Because micronuclei may result as well from mis-repaired and/or unrepaired DNA DSBs (Fenech et al., 2011; Ye et al., 2019) we decided to explore whether DSB repair is affected in *gip1gip2*. Firstly, DSBs were detected and quantified using the neutral comet assay. Our analyses were performed on isolated nuclei from *gip1gip2* seedlings showing a strong phenotype (Batzenschlager et al., 2015), the single mutants *gip1* and *gip2* and on nuclei of the corresponding WT controls (i.e., Col-0xWS, WS, and Col-0). Compared to their respective controls, comet tails appeared increased in both single and double *gip* mutants (Figure 1A). In order to quantify more precisely their respective genomic instability, DNA damage was evaluated upon visual scoring of each individual comet tail as previously described (Roa et al., 2009). While *gip1* and *gip2* presented about 1.4 times more DSBs than their respective WT control, *gip1gip2* showed 2.4 times more DSBs than Col-0xWS (Figure 1B). Altogether, our data suggest that *GIP1* and *GIP2* may have some overlapping functions to maintain genome stability and to limit DNA damages in interphase nuclei under normal growth conditions.

### Nuclear γ-H2AX Foci Are Highly Heterogenous in Size and Number in *gip1gip2*

**FIGURE 2:**
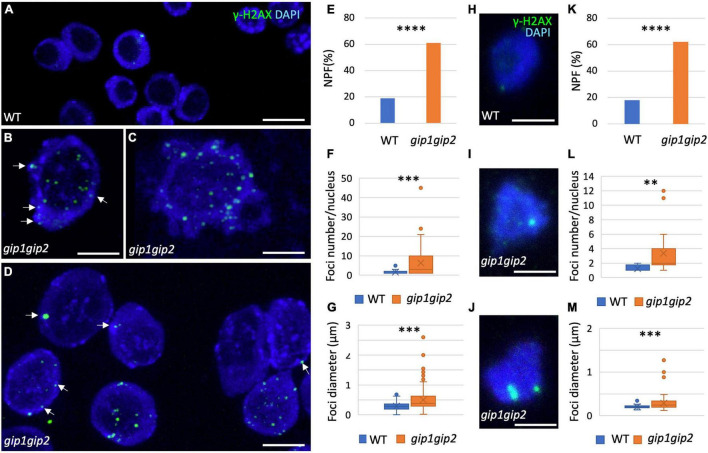
Characterization of the γ-H2AX foci in root meristematic nuclei. **(A–D,H–J)** Immunolocalization was performed on nuclei from squashed root tips of 9-day-old seedlings using specific anti-γ-H2AX antibody and DAPI staining in WT **(A)** and *gip1gip2* **(B–D)** as well as on 2C flow sorted nuclei from WT **(H)** and *gip1gip2* **(I,J)** root seedlings. Representative images are presented. White arrows indicate foci close to the bright DAPI-stained chromocenters. **(E–G,K–M)** Different characteristics regarding the γ-H2AX foci were quantified in WT (*n* = 249) and *gip1gip2* (*n* = 133) nuclei from root meristem nuclei and in 2C flow sorted nuclei from WT (*n* = 54) and *gip1gip2* (*n* = 61), such as the percentage of Nuclei Presenting Foci (NPF; **E,K**), the number of foci per nucleus **(F,L)** and the foci diameters **(G,M)**. Results were obtained from four independent roots. The Fisher’s exact test was used and the two-tailed-value is < 0.0001 (^****^) in panels **(E,K)**. The Mann Whitney test was used in panel **(F,G,L,M)** where *p-*values are indicated as < 0.01 (^**^) and < 0.001 (^***^). Scale bar = 5 μm.

To further analyze the significant increase of DSBs in *gip1gip2* and determine their localization at the cellular level in root meristematic nuclei, we performed immunolocalization with an antibody directed against γ-H2AX, a phosphorylated form of the H2AX histone variant known to be associated with sites of DSBs (Lang et al., 2012). Compared to the low occurrence of nuclei showing γ-H2AX foci in WT, the *gip1gip2* double mutant showed a significantly higher proportion of nuclei with foci and, frequently, several foci per nucleus (Figures 2A–D). The foci were found in the nucleoplasm, around the nucleolus and at the nuclear periphery. Among them, 22% were associated to the chromocenters (Figures 2B–D, see white arrows).

The γ-H2AX foci were observed in 19% of the nuclei in the WT control (hereafter named NPF for Nuclei Presenting Foci), while the proportion of NPF was 3.2 times higher in the double mutant (Figure 2E). Moreover, compared to the WT control in which a mean of 1 focus per nucleus was evaluated, the number of foci per nucleus was significantly increased in the double mutant, reaching a mean of 6 ± 1 foci/nucleus (Figure 2F). While the foci mean diameter was relatively homogenous in the WT control with a mean of 0.3 μm ranging from 0.1 to 0.7 μm, it appeared larger in the double mutant with a mean of 0.5 μm and strongly heterogeneous with diameters ranging from 0.2 to 2.5 μm (Figure 2G).

Previously, *gip1gip2* plants were described to display ploidy instability (Janski et al., 2012; Batzenschlager et al., 2015). To disentangle appearance of γ-H2AX foci from ploidy defects, we next focused our analysis on 2C flow sorted nuclei from root seedlings (Figures 2H–M). Proportions of NPF in the WT control (18%) and the *gip1gip2* double mutant (62%) (Figure 2K) were similar as for root meristematic nuclei (Figure 2E). In addition, both the number of foci per nucleus and their individual sizes were significantly higher in the double mutant compared to the WT control (Figures 2L,M). Indeed, WT nuclei with γ-H2AX associated signal presented rarely more than 1 focus, while in the double mutant the number of foci per nucleus could reach up to 12 (Figure 2L). Similarly, the diameter of the foci was rather constant in WT control nuclei, with an average around 0.2 μm, while in *gip1gip2* it varied between 0.12 to more than 1.2 μm with a mean diameter of 0.3 μm (Figure 2M). Interestingly, among large foci (*n* = 81; Figures 2I,J), 70% were detected at the nuclear periphery and 30% around the nucleolus. Smaller and less intense ones were distributed randomly through the nucleoplasm.

Consistent with the higher rate of DNA lesions measured by the comet assay, these results altogether indicate that *gip1gip2* over-accumulates γ-H2AX foci that are heterogenous in size. These foci are notably found at the nuclear periphery close to the chromocenters.

### Differential Activation of the Double-Strand Break Repair Pathways in *gip1gip2*

**FIGURE 3:**
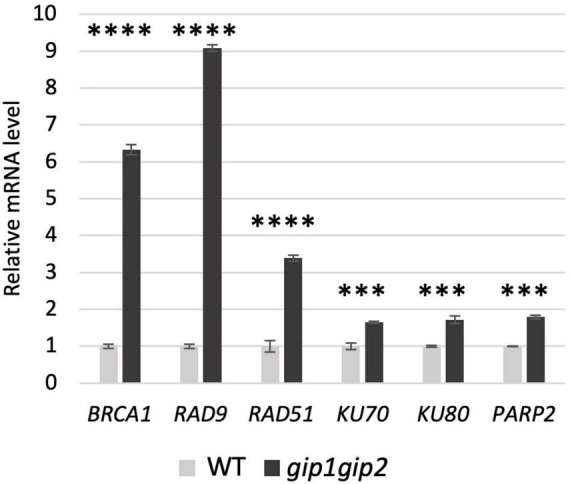
mRNA level of DNA damage responsive genes in *gip1gip2.* Relative mRNA level of selected DNA damage responsive genes in *gip1gip2* was compared to WT. Experiments of RT-qPCR was performed on RNA isolated from 9-day-old seedlings using specific set of primers (see Supplementary Table 1). Two independent experiments were performed. SDs are indicated. Unpaired *t* test was used where *p*-values are indicated as <0.0001 (^****^) and <0.001 (^***^), respectively.

Since we observed a constitutively high level of DSBs and numerous γ-H2AX foci in *gip1gip2*, we next investigated which DNA repair process was impaired. Firstly, we have checked the expression level of genes involved in the DSBs repair using quantitative real-time PCR (RT-qPCR). To this end, we selected genes encoding key factors involved in the two main DSB repair pathways: *(i)* the HR, restricted to cells in the S/G2 phase to resolve stalled replication fork and *(ii)* the non-homologous end-joining pathways (NHEJ) involved in repair of most DSBs (West et al., 2004). As representative of the HR pathway, we choose genes encoding HR regulators such as *BRCA1* (Lafarge and Montane, 2003) and *RAD9* (Heitzeberg et al., 2004), as well as the gene encoding the HR effector *RAD51* (Bleuyard et al., 2006). As indicative of the NHEJ pathway, *KU70* and *KU80* genes were analyzed for the classical NHEJ (c-NHEJ; Charbonnel et al., 2010), and the poly (ADP-ribose) polymerase encoding gene *PARP2* for the KU-independent backup-NHEJ (b-NHEJ) pathway (Jia et al., 2013). The expression of all tested genes was up-regulated in *gip1gip2*, indicating the activation of the DDR. However, contrary to those involved in NHEJ pathways, the genes representative of the HR pathway were the most induced in *gip1gip2* compared to WT (Figure 3). Indeed, *BRCA1* and *RAD9* encoding upstream regulators of HR, showed the highest fold change in their expression level, ranging between 6 and 9, respectively. The *RAD51* expression showed also a significant increase compared to WT with a 3.4-fold change, while the expression of NHEJ pathway genes (*KU70, KU80* and *PARP2*) remained only weakly induced (between 1.6- and 1.8-fold change).

**FIGURE 4:**
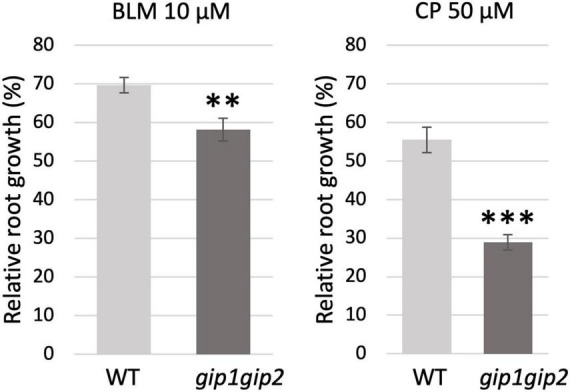
Sensitivity of *gip1gip2* toward drugs inducing DNA damage. Five-day-old seedlings grown on ½ MS media were transferred on ½ MS containing either 10 μM bleomycin (BLM) or 50 μM cisplatin (CP). Root growth was evaluated after 48 h on genotoxins in WT (n_*BLM*_ = 16, n_*CP*_ = 19) and *gip1gip2* (n_*BLM*_ = 20, n_*CP*_ = 28) (see Supplementary Figure 1). Percentages of relative root growth are presented. Two independent experiments were performed. SDs are indicated. The Fisher’s exact test was performed and *p*-values are indicated as < 0.01 (^**^) and < 0.001 (^***^).

In order to further determine which DNA repair pathway(s) were more specifically affected in *gip1gip2*, we next tested this double mutant in root growth assays in presence of the DNA damaging agents bleomycin (BLM) or cisplatin (CP). While the DSBs induced by BLM are classically repaired by both HR and NHEJ, the intra- and inter-strand DNA cross-links bridges induced by CP are preferentially repaired through HR during DNA replication (Fuertes et al., 2003). Therefore, we tested WT and *gip1gip2* root growth on media containing 10 μM BLM and 50 μM CP, respectively, as previously described (Roa et al., 2009; Biedermann et al., 2017). Contrary to WT plants in which root growth was similarly slowed down by BLM and CP, *gip1gip2* root growth appeared more affected upon CP treatment than upon BLM treatment (Figure 4 and Supplementary Figure 1). The higher sensitivity to CP (twice more compared to WT) than to BLM (decrease of only 1.2-fold) in *gip1gip2* is indicative of a predominant defect in HR repair.

Together, the major up-regulation of genes related to HR in *gip1gip2* and the increased sensitivity of the mutant to CP, suggest that GIP1 and GIP2 may be important to regulate genome maintenance through HR in somatic cells.

### RAD51 and γ-H2AX Foci Rarely Colocalized in *gip1gip2* Nuclei

**FIGURE 5:**
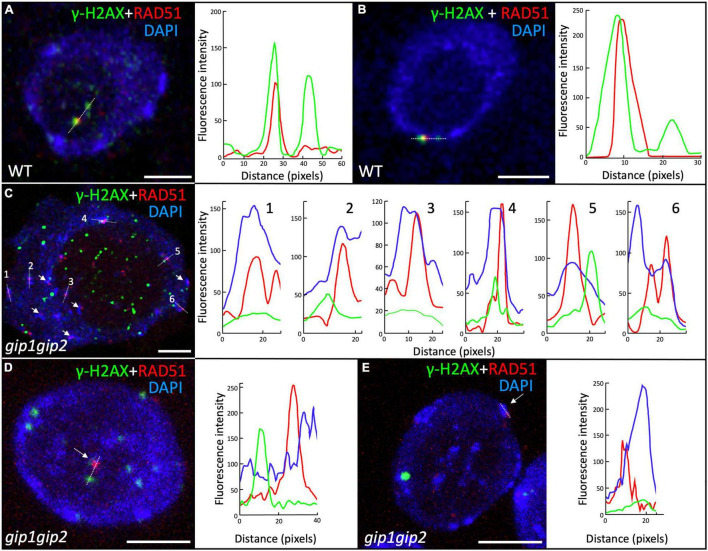
Localization of the γ-H2AX and RAD51 foci in root meristematic nuclei. Immunolocalization was performed on 9-day-old seedlings root tip nuclei using antibodies against γ-H2AX and RAD51 together with a DAPI staining. Representative images of γ-H2AX and RAD51 foci in WT **(A,B)** and *gip1gip2* **(C–E)**. Fluorescence profiles beside **(A,B)** illustrate the colocalization of γ-H2AX (green) and RAD51 (red) foci along the white dotted lines in WT. White arrows indicate RAD51 foci located at or close to bright DAPI-stained chromocenters in *gip1gip2*. Fluorescence profiles for γ-H2AX (green) and RAD51 (red) foci as well as high DAPI intensity for chromocenters (blue) are presented along the numbered white dotted lines indicated on the panels **(C–E)**. This highlights that RAD51 foci are located at/or very close to chromocenters in *gip1gip2*. Scale bars = 5 μm.

**FIGURE 6:**
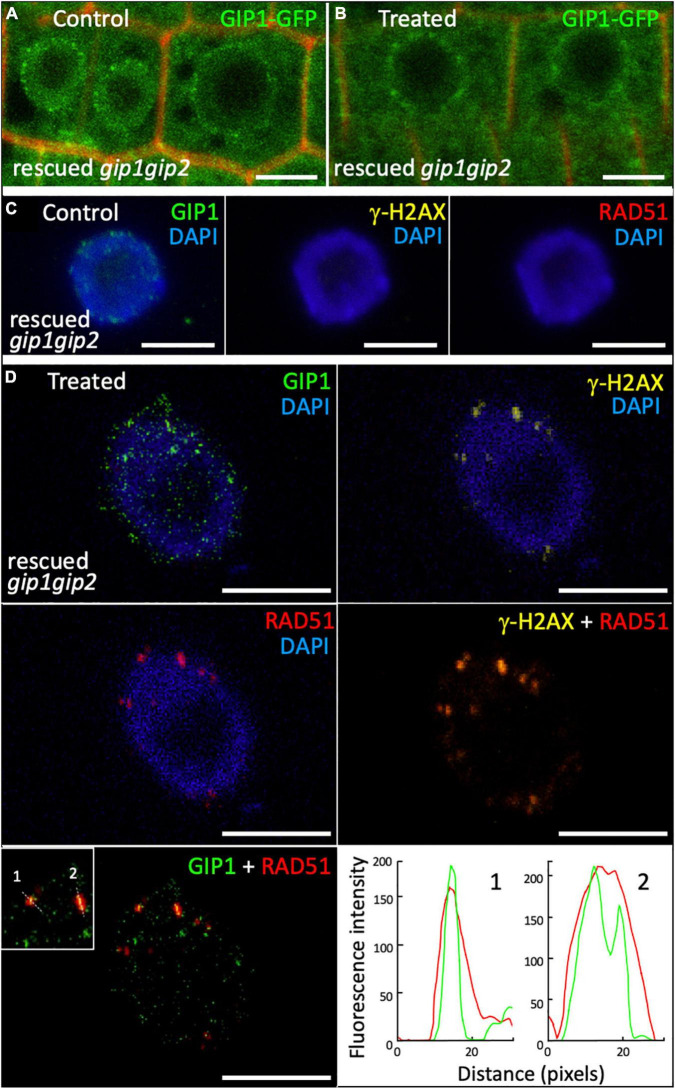
Localization of GIP1-GFP in response to DNA damage induced by cisplatin. *GIP1-GFP* was expressed in the *gip1gip2* mutant and rescued cellular phenotypes as described (Batzenschlager et al., 2015). Nine-day-old seedlings were treated or not (control) by 50 μM CP during 16 h in ½ MS. **(A,B)** GIP1-GFP localization was analyzed in root meristematic nuclei in control **(A)** and treated **(B)** cells. Cell wall was detected by propidium iodide staining. Representative images are presented. **(C,D)** Immunolocalization was performed on root tip nuclei using antibodies against GFP, γ-H2AX and RAD51 and DAPI staining. Two independent experiments were performed. Representative images of GIP1-GFP, γ-H2AX, and RAD51 foci are presented for control **(C)** and treated **(D)** cells. Merge images are presented for γ-H2AX (yellow) and RAD51 (red) as well as for GIP1-GFP (green) and RAD51 (red). Fluorescence profiles illustrate the colocalization of GIP1-GFP (green) and RAD51 (red) foci along the white dotted lines indicated on the left panel. Scale bars = 5 μm.

In interphase nuclei from root meristems, the *gip1gip2* mutant accumulated a high number of γ-H2AX foci. This is indicative of defects in DNA repair, notably through HR, as shown by the sensitivity of the mutant to CP. Thus, we further investigated the nuclear distribution of the HR effector RAD51. We performed co-immunolabeling on WT and *gip1gip2* root tip nuclei using together specific antibodies against RAD51 and γ-H2AX (Figure 5). As expected, a few nuclei showed RAD51 foci in WT (5.5%, *n* = 109) which colocalized systematically with γ-H2AX foci (Figures 5A,B) as described previously (Biedermann et al., 2017). Such colocalization is further supported by fluorescence profiles presented in Figures 5A,B. In agreement with the increased number of γ-H2AX foci (Figure 2), the number of RAD51 foci was also significantly increased in *gip1gip2*, with 36% of nuclei showing RAD51 foci with an average of four per nucleus. Interestingly, the number of RAD51 foci per nucleus was significantly lower than the number of γ-H2AX foci and surprisingly most of the RAD51 foci showed no clear colocalization with γ-H2AX foci in *gip1gip2* (73%, *n* = 224) (Figures 5C–E). In addition, 61% of the RAD51 foci were mainly found at/or very close to the bright DAPI-stained chromocenters (Figures 5C–E, see white arrows) without the presence of γ-H2AX or no clear colocalization with γ-H2AX. Since a substantial decrease in the activity of GIP1 and GIP2 in the double knockdown mutant *gip1gip2* may impair RAD51 and γ-H2AX colocalization, we further explored the localization of GIP1-GFP upon genotoxic stress using CP. Using live cell imaging, GIP1-GFP in the nucleus appeared to be located close to the NE, as previously described (Janski et al., 2012; Batzenschlager et al., 2013; Batzenschlager et al., 2015) and this location was maintained when seedlings were treated by 50 μM CP for 16 h (Figures 6A,B). As expected, compared to the control (Figure 6C), we observed that all RAD51 foci induced by CP colocalized with γ-H2AX foci using immunolabeling (Figure 6D, merge RAD51 and γ-H2AX), while 23% (*n* = 444) of the RAD51 foci also colocalized with GIP1-GFP at the nuclear periphery (Figure 6D, merge RAD51 and GIP1-GFP with corresponding colocalization profiles).

Here, we showed that RAD51 and γ-H2AX foci are mostly not colocalizing in *gip1gip2* and that RAD51 foci are surprisingly located more frequently at chromocenters in the mutant. Interestingly in response to CP, RAD51, besides being systematically colocalized with γ-H2AX foci in the complemented mutant, partly colocalizes with GIP1-GFP at the nuclear periphery.

## Discussion

In the present study we have investigated the genomic instability in the *gip1gip2* mutant which showed an increased DSBs occurrence compared to the WT control. Besides the constitutive activation of the HR pathway, the mutant presented a stronger sensitivity to the genotoxic agent cisplatin, indicative of an impaired HR in root meristematic cells. This may rely on the absence of a clear expected colocalization between RAD51 and γ-H2AX foci in the mutant. Thus, we highlight novel functions of GIPs in the maintenance of genome stability at the nuclear periphery.

### How Genome Instability Occurs in *gip1gip2*

At the cellular level, numerous γ-H2AX foci are detected in *gip1gip2* and their heterogenous size may reach up to 2.5 μm in diameter (Figure 2), indicating a permanent unrepaired DNA damage as described (Costes et al., 2006). This is contrasting with the low number of γ-H2AX foci which are smaller and more homogenous in size in WT, where active DNA repair occurs. As the mutant is mostly sensitive to cisplatin, this is indicative of a defective HR repair during DNA replication, leading to the accumulation of DSBs. Some of the γ-H2AX foci in *gip1gip2* are found at the nuclear periphery close to the chromocenters, which are mainly constituted by repetitive sequences and pericentromeric heterochromatin (Fransz et al., 2002). As centromeric heterochromatin organization was affected in *gip1gip2* (Batzenschlager et al., 2015), we cannot exclude defect in heterochromatin replication coupled with defective DNA repair. In this respect, we observed twice more late S-phase replicating nuclei in *gip1gip2* compared to WT (Supplementary Figure 2A), with the presence of γ-H2AX foci in the vicinity of late S-phase replicating DNA, mainly corresponding to pericentromeric heterochromatin (Supplementary Figure 2B). This defect in DNA repair could also explain the accumulation of G2-stalled 4C nuclei in *gip1gip2* (Janski et al., 2012; Batzenschlager et al., 2015). Alternatively, unscheduled RAD51 removal from DNA may also lead to its overaccumulation at stalled replication forks, as described in USO2 cells (Parplys et al., 2015). Thus, such overaccumulation leads to a decreased HR efficiency and an increased genomic instability. Together, besides the activation of the HR pathway, defects in DNA replication as well as abnormal RAD51 loading may result in the increased genomic instability observed in *gip1gip2*.

### Importance of the Nuclear Periphery for Genomic Maintenance in Plants

In plants, γ-H2AX form foci at DSB sites, where HR proteins such as RAD51 and RAD54 are sequentially recruited (Biedermann et al., 2017; Horvath et al., 2017; Meschichi et al., 2021). Moreover, RAD54 forms foci at DSBs that are restricted to S and G2 phase cells in roots (Hirakawa et al., 2017) and localized at the nuclear periphery close to the nuclear envelope at high frequency (Hirakawa and Matsunaga, 2019). Interestingly, this peripheral location was reduced in the double mutant for the plant nucleoskeleton components CRWN1 and CRWN4 after γ-irradiation (Hirakawa and Matsunaga, 2019). Furthermore, an interaction between RAD51 and RAD54 was already detected in plants (Klutstein et al., 2008) and RAD54 may contribute to the removal of RAD51 at replication forks, as demonstrated in human cells (Mason et al., 2015). Together, we cannot exclude that, like for RAD51 which partially colocalized with GIP1 (Figure 6D), the function of RAD54 might also be affected in *gip1gip2* due to its possible impaired localization. Moreover, considering the crucial role of NPCs for DNA repair in animal and yeast (Freudenreich and Su, 2016), defects in the shape and the distribution of NPCs reported in *gip1gip2* (Batzenschlager et al., 2013) may also partially explain the defective HR repair we observed here. Together, GIP proteins may contribute to the spatio-temporal recruitment of RAD51 for efficient HR in genomic maintenance at the nuclear periphery.

### GIP at the Crossroad of DNA Repair and Cell Cycle Regulation

As GIPs are important to recruit microtubule (MT) nucleation complexes (Janski et al., 2012), we cannot exclude a role of either MTs or the γ-tubulin complex in the regulation of DNA repair. Indeed, MTs connected to the LINC complex were described to induce chromatin mobility around DSBs to promote efficient DNA repair in mammals (Lottersberger et al., 2015). Alternatively, an additional specific intranuclear function of GIPs and some of the MT nucleation complex proteins such as γ-tubulin, independent of MT dynamics and/or MT nucleation, may exist as well. Indeed, plant γ-tubulin was found at the inner membrane of NE in association with SUN1 (Chumova et al., 2019), and GIPs are also located on both sides of the NE (Batzenschlager et al., 2013; Batzenschlager et al., 2014).

GIPs are found at the nuclear periphery close to chromocenters (Batzenschlager et al., 2015). Interestingly, RAD51 localization at DNA lesions is mediated by RBR1 (Biedermann et al., 2017) which can form foci with γ-H2AX in the close vicinity of chromocenters (Horvath et al., 2017). In addition, both RAD51 and GIPs were shown to be substrates of the cell cycle regulators B1-type cyclin complexes (Motta et al., 2021), which activity drives the recruitment of RAD51 at DNA damage sites (Weimer et al., 2016). In line with this and similarly as our report on *gip1gip2*, *cycB1* mutants are mainly sensitive to cisplatin (Weimer et al., 2016). Altogether these data suggest that a dynamic crosstalk may involve GIPs as well as the cell cycle regulators RBR1 and B1-type cyclin complexes to allow proper loading of RAD51 at DNA lesions. However, the underlying mechanisms need to be further explored.

## Conclusion and Outlooks

Our data reveal an interesting role of GIPs at the nuclear periphery in the cross-talk between DNA replication and DNA repair in connection with chromatin organization. Indeed, our previous work had shown a synergism between GIPs and BRUSHY1 (BRU1)/MGOUN3 (MGO3)/TONSOKU (TSK) to maintain centromeric cohesion (Batzenschlager et al., 2017). BRU1 was also suggested to play a role in the structural and functional maintenance of chromatin during DNA replication (Takeda et al., 2004; Suzuki et al., 2005). Nowadays, we need to further explore the role of both GIPs and BRU1 in the genomic maintenance at the nuclear periphery during DNA replication. Moreover, besides the abnormal shape of *gip1gip2* nuclei, we cannot exclude a rupture of the NE as a result of mechanically stressed nuclei (Goswami et al., 2020). Indeed, more rigid nuclei were described as defective in nuclear mechanics and thus triggering DNA damage in animals (Nava et al., 2020). This also opens the way to investigate the link between DNA repair and nuclear mechanics in plants.

## Data Availability Statement

The original contributions presented in the study are included in the article/Supplementary Material, further inquiries can be directed to the corresponding author.

## Author Contributions

M-EC: conceptualization, writing – original draft, and resources. M-EC and EHE: methodology. GS, MB, GH, EHO, DT, M-EC, and A-CS: investigation. AB, M-EC, GS, and MB: writing – review and editing. M-EC and AB: funding acquisition and supervision. GS, AB, and M-EC: figure preparation. All authors contributed to the article and approved the submitted version.

## Conflict of Interest

The authors declare that the research was conducted in the absence of any commercial or financial relationships that could be construed as a potential conflict of interest.

## Publisher’s Note

All claims expressed in this article are solely those of the authors and do not necessarily represent those of their affiliated organizations, or those of the publisher, the editors and the reviewers. Any product that may be evaluated in this article, or claim that may be made by its manufacturer, is not guaranteed or endorsed by the publisher.
