# Supplementary material for: GIP1 and GIP2 Contribute to the Maintenance of Genome Stability at the Nuclear Periphery
Source: Front Plant Sci. 2022 Jan 27;12:804928. doi: 10.3389/fpls.2021.804928 (PMC8830487; doi:10.3389/fpls.2021.804928)
Supplement: Supplementary file 1 [file Presentation_1.pdf]

Supplementary information

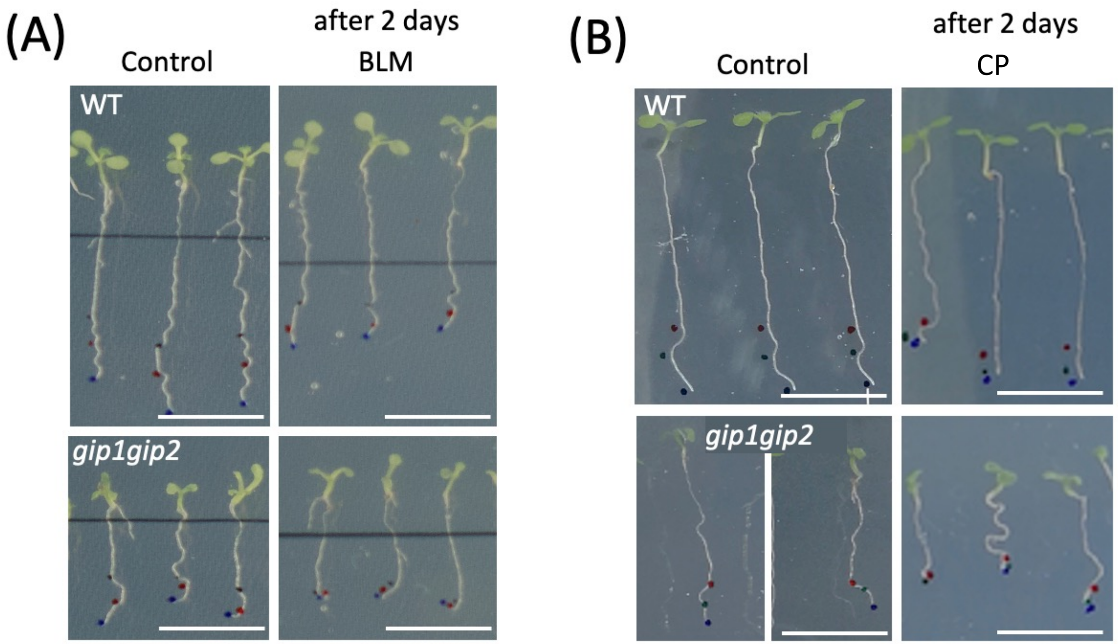

Figure S1: Sensitivity of *gip1gip2* seedlings to bleomycin and cisplatin. Five day-old-seedlings were transferred on media containing either 10 μM bleomycin (BLM) (A) or 50 μM cisplatin (CP) or 1/2MS (control). After 2 days on BLM (A), the root growth of the mutant was slightly reduced compared to WT, but in presence of cisplatin (B) it was considerably affected. Scale bar = 1 cm.

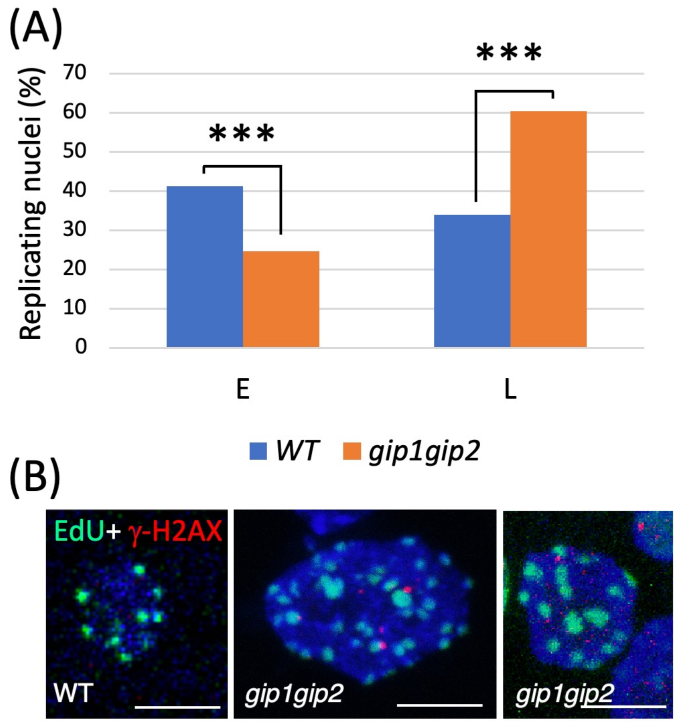

Figure S2: Analysis of replicating nuclei in early and late S phases. (A) EdU labelling was performed on 9-day-old seedlings for 90 min as described (Noir et al., 2015) and quantification of early (E) and late S-phases (L) nuclei was performed in WT (n=262) and in *gip1gip2* (n=162) after root squashing. Fisher's exact test was used and the two-tailed –value is <0.0001 (***). (B) After EdU labelling (green), immunocytochemistry was performed on WT and *gip1gip2* root tip meristems using anti-g H2AX antibody (red) and DAPI staining (blue). Scale bar= 5 μm.

Supplementary Table S1

List of forward (F) and reverse (R) primers used in RT-qPCR experiments

BRCA1	F: CCATGTATTTTGCAATGCGTG<br>R: TGTGGAGCACCTCGAACTTCT
RAD9	F : CCCGAGTACGGGTACAGAAC<br>R: CATTTGCATCCACCCAATTACTAGG
RAD51	F: CGAGGAAGGATCTCTTGCAG<br>R: GCACTAGTGAACCCCAGAGG
KU70	F: GGTGACGCAGATGATGGAAA<br>R: TGTCTTCAGTTCGACCACAGTCA
KU80	F: TCGTAAAGGCTGCGTCTTGG<br>R: TTGGCATAACTTGAATAGATGGTTCA
PARP2	F: ACTGTCTACGATACAGCCCAGGTG<br>R: TGGTTCAGGCTCATCTCTTGTGC
